# Information and Communications Technology–Based Interventions Targeting Patient Empowerment: Framework Development

**DOI:** 10.2196/17459

**Published:** 2020-08-26

**Authors:** Liran Karni, Koustuv Dalal, Mevludin Memedi, Dipak Kalra, Gunnar Oskar Klein

**Affiliations:** 1 Centre for Empirical Research on Information Systems Örebro University School of Business Örebro Sweden; 2 School of Health Sciences Mid Sweden University Sundsvall Sweden; 3 European Institute for Innovation through Health Data Gent Belgium

**Keywords:** empowerment, ICT intervention, digital health, eHealth, framework model, ICT patient empowerment model (ICT4PEM)

## Abstract

**Background:**

Empowerment of patients is often an explicit goal of various information and communications technology (ICT) (electronic, digital) interventions where the patients themselves use ICT tools via the internet. Although several models of empowerment exist, a comprehensive and pragmatic framework is lacking for the development of such interventions.

**Objective:**

This study proposes a framework for digital interventions aiming to empower patients that includes a methodology that links objectives, strategies, and evaluation.

**Methods:**

This study is based on a literature review and iterated expert discussions including a focus group to formulate the proposed model. Our model is based on a review of various models of empowerment and models of technology intervention.

**Results:**

Our framework includes the core characteristics of the empowerment concept (control, psychological coping, self-efficacy, understanding, legitimacy, and support) as well as a set of empowerment consequences: expressed patient perceptions, behavior, clinical outcomes, and health systems effects. The framework for designing interventions includes strategies to achieve empowerment goals using different ICT services. Finally, the intervention model can be used to define project evaluations where the aim is to demonstrate empowerment. The study also included example indicators and associated measurement instruments.

**Conclusions:**

This framework, which includes definitions, can be useful for the design and evaluation of digital interventions targeting patient empowerment and assist in the development of methods to measure results in this dimension. Further evaluation in the form of interventional studies will be needed to assess the generalizability of the model.

## Introduction

Information and communication technologies (ICT) have already transformed all aspects of health care and, along with technological developments and innovation, ICT will continue to strengthen its role in the future. ICT development initially targeted health professionals as primary users through the provision of electronic health record systems [1]. Recently, however, patients have emerged as additional primary users and targets of ICT interventions. The most abundant examples of this strategy are ICT interventions targeting patient communication (unidirectional or bidirectional), such as messaging, chat services, and real-time video meetings that replace personal contact with physicians or nurses. The social media revolution has led to online patient communities and networks with empowering effects on patients through the exchange of information and group representation [2]. Providing patients with access to their own personal health records and various online patient education programs has been widely viewed by stakeholders as a tool for patient empowerment [3-5]. ICT-based strategies have also grown in popularity and proved to be efficient in the self-administered management of various conditions such as online cognitive behavioral therapies for depression and anxiety [4,6,7]. Recent efforts include ICT interventions in the form of home-based sensors, wearable devices, and mobile apps for empowering both patients and their physicians by providing accurate and detailed information about disease symptoms and management [8]. The evolving concept designated as quantify-self, along with artificial intelligence and machine learning, harbors as of yet largely unexplored potential in health care.

The concept of patient empowerment is closely associated with the ongoing paradigm shift from a paternalistic to a patient-centered model of engagement. In recent decades, health care systems have undergone a major paradigm shift from a patient-doctor relationship towards a patient-health care relationship where patients, as consumers, are enjoying more equality in the provision of, and access to, their own health care [9,10]. Decision makers, including the World Health Organization, have long considered patient empowerment as a priority, based on the anticipation that it can translate into widespread improvements of disease control, optimized health care utilization, and patient satisfaction. Expectations are high for ICT being capable of improving patient empowerment, self-efficacy, and self-assessment. Accordingly, numerous ICT interventions [5] have claimed to enhance patient empowerment. It is therefore surprising that scientific evidence supporting this notion is rather limited [11]. Although there is general agreement that patient empowerment conceptualizes the enablement of self-control and self-efficacy, its exact boundaries, content, and operationalization have remained elusive and are variably described by published frameworks of the concept [12,13]. Through a lack of unifying definitions, studies that include assessments of patient empowerment often apply arbitrary definitions or simply leave the concept undefined. Studies of patient empowerment usually include components of other potentially associated parameters, such as disease control, quality of life, and health care utilization, as descriptors or integrated parts of patient empowerment. The methodological and conceptual controversies, including patient empowerment assessment, as recently reviewed [14,15], explain and are paralleled by the current inability to reliably measure patient empowerment.

There is a clear demand for further investigations to understand how ICT interventions may affect patient empowerment and alter other important related parameters. However, there is no model that links a conceptual analysis of empowerment to strategies for ICT interventions and evaluation.

Studies that aim to intervene and assess patient empowerment often arbitrarily conceptualize, or simply refrain from providing, a specific definition for empowerment. This makes the interpretation and comparability of these studies difficult and further contributes to conceptualization-related obscurities.

The objective of this study was to develop a framework called ICT for Patient Empowerment Model (ICT4PEM) for ICT interventions that aim to empower patients. The framework provides a methodology to link objectives, strategies, ICT services, and evaluation. Precise definitions of the terms used in the model of empowerment were developed, and examples of indicators to be used for its evaluation are provided.

## Methods

This study applies multiple methodologies including a scoping literature review for laying down the theoretical foundation for the ICT4PEM framework, followed by various forms of iterative expert discussions to finalize the concepts of the framework elements. A focus group discussion (FGD) was conducted to review, modify, and finalize the ICT4PEM framework. The utility of the framework was further demonstrated in case studies where patient empowerment had been an explicit objective.

### Scoping Review

We started by conducting a scoping review in accordance to the recommendations by Levac et al [16], to identify the relevant literature and knowledge gaps and to establish a theoretical foundation with respect to our research purpose, which was to identify conceptual and methodological issues and necessary requirements for conducting meaningful ICT interventions targeting patient empowerment. A scoping review is effective for identifying a knowledge gap, scoping the body of literature, and clarifying concepts [17].

To identify the questions for researching the concepts of ICT intervention and patient empowerment, we searched for review articles in PUBMED, using the following combinations of search terms: “ICT” OR “eHealth” AND “patient empowerment” AND “Intervention“ AND “Evaluation.” A group of 4 of the authors identified and selected relevant studies by first reading the article titles and then reading abstracts. Every researcher individually read and proposed relevant articles based on our scope and the objective of the study. Then, in a decision-making meeting, all 4 researchers presented their proposed articles. At the meeting, the articles relevant to the objective and research questions were selected through consensus.

We also used the snowball technique to retrieve additional relevant articles [18]. The inclusion of relevant studies with respect to the research question and final purpose was based on the assessment of methodological and contextual relevance through regular meetings. One junior and one senior researcher mapped, summarized, and classified the information in these articles. Potential disagreements were resolved by a third, senior researcher. We charted the information based on the starting points of initial study identification with subsequent iterative subgrouping, in accordance with the accumulating knowledge regarding the research question and purpose.

The analyses did not attempt to include all of the existing literature on this concept, which has been used for many different types of discourses. Even if definitions for various characteristics and measurement instruments of empowerment were searched broadly, the main scope that guided the review and analyses was the use of ICT to empower individual patients. Therefore, aspects of empowerment of all of the patients as a political group vs. society or health professionals were excluded. Results of the scoping review were summarized and applied as the input and theoretical foundation for the authors’ subsequent iterative step, as well as for expert consultations as described in the following sections.

### Expert Discussions

The initial draft of the new framework with the inclusion of the initial variables of interest was subsequently discussed in a series of consultations with domain experts, in order to refine the elements of the framework. This involved a series of iterative feedback rounds with experts in electronic health (eHealth), health and implementation science, and patient empowerment*.* Input from the experts was then integrated into the framework through parallel and iterative rounds of consensus-seeking consultations between the authors, followed by presentations of our draft model at various professional conferences including the eHealth conference Vitalis in Gothenburg, Sweden, May 2019 and Swedish DOME consortium meeting in Karlstad, Sweden, June 2019 and was published as “research in progress” at the AMCIS conference [19] allowing the model to be refined through multiple steps.

### Focus Group Discussion

The emerging version of ICT4PEM was further evaluated and modified based on an FGD. The FGD involved 8 participants from Germany, Spain, the United Kingdom, and Sweden. The participants were health care professionals, health systems researchers, and informatics specialists. Due to geographical constraints, the FGD was conducted online using the ZOOM platform and lasted 120 minutes. All the FGD participants received a brief written description of the ICT4PEM a week prior to the meeting. The FGD started with a short presentation of the framework. The FGD objectives were to discuss the design choices, content, and perceived usefulness of the framework. A small number of prompts were used to elicit discussion. The FGD was recorded with due permission from the participants. One senior researcher also took notes during the FGD.

The entire recording of the FGD was transcribed. Qualitative content analysis was conducted, following the guidelines by Graneheim et al [20]. The participants’ words were analyzed as the actual content; hereafter, the interpretation and judgment of participants’ responses were analyzed as latent content [21]. We analyzed the data with a repeated look over the written transcription by identifying each of the units of meaning and listening to the audio recording [20]. After the analysis and judgments, we modified and finalized the ICT4PEM framework.

### Case Study Examples (Demonstration)

The resulting framework was demonstrated by applying it to 2 of our recent projects in eHealth as examples where empowerment had been an explicit objective: the EU-project C3-Cloud and Swedish project EMPARK.

## Results

### Theoretical Foundation for Developing the ICT4PEM Framework

#### ICT Intervention-Specific Requirements for the Conceptualization of Patient Empowerment

The conceptual obscurity of the boundaries of this concept, as well as the lack of widely accepted definitions for its included conceptual elements, were identified during the scoping review process as major obstacles for designing, implementing, and evaluating ICT interventions for patient empowerment.

It is important to recall that empowerment as a concept, in general, emerged as a descriptor of a mental state or the process leading to such a mental state in a group of individuals [22]. The approach to try to understand and describe patient empowerment as a mental state by conducting qualitative patient interviews [23] is especially compelling in this regard and may also help to circumvent the conceptual uncertainties [12,24] related to the wide variety of different scholarly definitions. Moreover, patient-derived descriptions of patient empowerment reveal a remarkable pool of internal (patient-perceived) dimensions of patient empowerment, which are consistent among the different studies despite their contextual (disease-specificity, health care, and social background) discrepancies. Behavioral (ie, patient engagement) or other health or health care parameters (ie, quality of life [QoL]) that are in presumed consequential relationships with the perception of internal empowerment fall outside of such a patient-centric patient empowerment paradigm, despite the fact that these parameters are often part of a wider patient empowerment paradigm in other models. The foremost advantages of such a patient-centric empowerment conceptualization become clear when ICT interventions on patient empowerment target the patients. This conceptualization enables the empowerment specificity of the intervention from the patient perspective, while also allowing for an analysis of the possible links between the targeted empowerment characteristics and secondary changes in other health care parameters.

Based on these considerations, 3 major conceptual requirements were identified, which were used as foundational grounds for developing the framework, including (1) patient-centeredness in the definition of boundaries and content for the concept of patient empowerment, (2) providing a clear distinction between patient empowerment defined as patient-derived perceptions and the consequential domains, and (3) clear definitions for each conceptual element.

#### Specific Requirements for ICT Interventions and Evaluation Targeting Empowerment

Most ICT interventions in health care are multifaceted with respect to their composition of incorporated ICT tools and methodologies. This multimethod approach of ICT interventions and the use of additional methods of interacting with participants have been shown to increase the effect of the ICT intervention. The literature also describes that a lack of conceptual constructs underlying the interventional target confers negative effects on intervention efficacy. Indeed, specific targets for the interventional steps may remain ill-defined across studies [25,26]. Specifically, ICT interventions for achieving patient empowerment are typical examples of this problem [26].

Therefore, the following requirements were identified and subsequently utilized during the framework design: (1) ICT interventions with the primary target of patient empowerment should have their theoretical foundation in an integrated framework that includes a clear conceptualization of patient empowerment, (2) ICT interventions should define empowerment as the primary target of intervention, and (3) the linkage of individual ICT services should correspond with target elements among the patient empowerment characteristics for the scientific quality, evaluability, reproducibility, and comparability of the intervention.

### The ICT4PEM Framework for ICT-Based Interventions Targeting Patient Empowerment

#### The Patient Empowerment Model (PEM)

**Figure 1:**
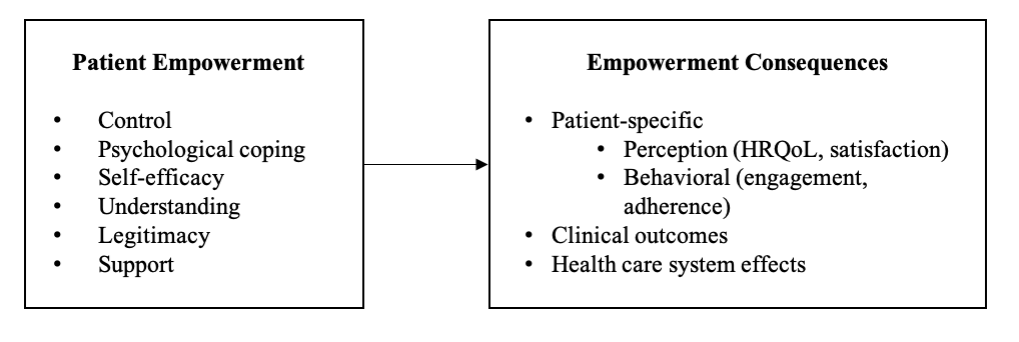
Patient empowerment model (PEM). HRQoL: health-related quality of life.

Figure 1 presents the patient empowerment model (PEM) and depicts the core characteristics of patient empowerment in the left box, while the right box depicts the possible consequences of empowerment that may result from the change in characteristics. The link between the two boxes indicates an indirect effect of empowerment on the consequences, since the latter can be affected by other types of interventions. For a specific ICT intervention, the project management selects the characteristics and consequences that are considered appropriate to study and be influenced. One should also view the attributes depicted here as the most important aspects of empowerment and its consequences, and it may be relevant to add further aspects in a specific case.

Although the composition of the patient empowerment conceptualization is unique and adapted to the necessities of interventional trial design, the individual conceptual elements of the model and their association with patient empowerment are derived from the literature. Based on the previously described conceptual considerations (see the section Theoretical Foundation for Developing the ICT4PEM Framework), constructs of PEM were selected based on qualitative studies aiming to understand the patient perspective in patient empowerment. The review performed by Agner et al [23] was used as a basis to identify conceptual elements that are common across the incorporated studies, irrespective of a specific disease or health care background. This selection consequently represents the core concept of perceived patient empowerment. Based on the findings by Agner et al [23], knowledge, control, and coping related to the disease and the process of health provision were selected along with the feeling of support and legitimacy, as explained in detail in Table 1.

**Table 1:** Characteristics of empowerment.

Characteristic	Definition
Control	ability by which an individual can decide about his or her level of engagement in the health care process and participate in decisions regarding alternative treatment options, also when these are performed by professionals Note: Patient control is described by [27] as “having the opportunity to use power, namely, making choices, implementing intentions, taking action, and affecting the actions of others”. Our definition of control is similar, though we consider control to be an ability, rather than an opportunity, as the latter suggests an imbalanced power situation between the provider and the patient.
Psychological coping	state of a process in which one psychologically tries to adapt to the challenges associated with the negative changes of health status Note: Coping strategies are traditionally divided into problem-focused coping (managing or altering the problem) and emotional-focused coping (regulating the emotional response to the problem) [28]. Our definition encompasses both these perspectives.
Self-efficacy	sum of cognitive and physical capabilities possessed by the patient that can be used for self-care Note: Köhler et al [29] stated that self-efficacy influences a patient on his/her thinking, feeling, motivation and his/her action towards an attempt of new health behavior.
Understanding	potential use of the information a patient has regarding his or her own *health status,* the *diseases*, and the function of the actual and possibly available *health care processes* Note: Understanding, in our definition, represents the patient´s capacity to apply *knowledge* in the specific and individual context of the disease and healthcare provision. *Information* and its availability to the patient serve as a base for understanding. Consequently, neither knowledge, nor information are sufficient as characteristics of *empowerment*, though both should be considered as pre-requisites for understanding.
Legitimacy	perception that the care from a professional health care system is fair with regard to issues of being lawful in the jurisdiction and available with a sufficient degree of equity Note: Legitimacy refers to the patient´s perception of fairness and trust in the healthcare system in general or in a specific situation. This is particularly important when the care situation may include aspects that are beyond the direct control of the patient, such as when a person is unconscious. The second aspect is associated with the perception of the right to receive required services to the same degree as other persons.
Support	quantity and quality of support as assessed by an individual of support that is being offered or received from the care provider or the non-medical supporting environment Note: Support can both enhance and decrease autonomy. Elderly, sick individuals often receive high amounts of unsolicited help that, although normally well-intentioned, may further reduce their possibility of making choices, autonomy, self-esteem and their longer-term competence or coping abilities [30].

We followed the general principles of the International Organization for Standardization (ISO) when constructing the definitions [31]. Thus, a definition is a single phrase that can replace the term wherever used and does not start with an article (eg, “a”, “the”) or end with a full stop.

Possible consequences of patient empowerment are various. The literature identifies several of these concepts either in connection to or as part of patient empowerment. Although their connections to the other conceptual dimensions of patient empowerment were mapped using the patient empowerment model of Bravo et al [12], PEM (Figure 1) rather considers them as part of an extended empowerment concept. Patient empowerment consequences are divided into 4 major groups: patient perceptions with close conceptual relationship to empowerment perception, (health-related quality of life [HRQoL] and patient satisfaction); behavioral elements (patient engagement and adherence); and finally, clinical outcome and health care system effects as their own categories, with definitions given in Table 2.

**Table 2:** Characteristics of empowerment consequences.

Characteristic	Definition
Health-related quality of life (HRQoL)	impact of health status on a person’s quality of life assessed with a multidimensional instrument Note 1: It is measured in general by EQ-5D [32] or SF-36^a^ [33]. Note 2: There are also disease-specific HRQoLs available (eg, the PDQ8^b^ for Parkinson’s disease [34].
Patient satisfaction	degree of fulfilment of the patient’s expectation of the health care services received Note 1: Patient satisfaction has been the subject of research and a fundamental driving force behind the health care policy development for decades. Satisfaction as a concept shows a tight linkage to the concept of expectations [35]. Note 2: Indicators may measure timeliness, effectiveness, and patient-centeredness as well as quality of health care staff including their interpersonal communication skills. In general, it may also include patient views on accessibility and the state of health care facilities. Note 3: There are several validated instruments for measuring patient satisfaction (eg, PSQ-18^c^ [36], GS-PEQ^d^ [37] and SAPS^e^ [38]). However, these instruments are non-generic and context-dependent.
Patient engagement	degree to which the patient is an active agent for managing their own health Note 1: This involves actions and collaborative partnerships at complex levels including the individual, familial, organizational, and health care policy levels and often in response to the recommendations of the health care professional system. Note 2: Studies assessing health care performance on patient engagement are few in number and limited by the lack of instrument able to assess it [39]. Recently, Graffigna et al [40] made efforts towards a conceptualization followed by the development and validation of a construct (Patient Health Engagement Scale) for patient engagement. More recently, researchers introduced another validated, 20-item construct, the Patient Engagement Index (PEI) for assessment [39].
Adherence	degree to which the patient’s behavior follows a care plan agreed to by the health professional and the patient Note 1*:* The care plan may not be an explicit document but a mutual understanding of a professional recommendation. Note 2: Patient adherence as a concept has practically replaced *compliance* with the evolution of the patient’s role in health care from being a passive, obedient recipient of a physician's authority to an active partnership. Our definition is in line with the WHO’s^f^ definition, which describes patient adherence as “the extent to which a person's behavior — taking medication, following a diet, and/or executing lifestyle changes — corresponds with agreed recommendations from a health care provider.”
Clinical outcomes	professional measurable health state of a patient allowing for a comparison before and after a health care intervention Note: The measurable indicator is often dependent on laboratory analysis or chemical, microbiological, physiological, or medical devices used or at least controlled by the health professional organization. However, clinical outcome can also be measured using some form of professional standardized clinical assessment following a defined process of observation. Further, patient-reported outcome measures can be regarded as clinical outcomes, provided they are collected in a way that is professionally validated.
Health care system effects	health care system resource utilization before, during, and after a specific new procedure such as an ICT^g^-based patient empowerment intervention Note: Here, we consider both productivity and quality, which can also be described as the degree to which the goals of the organization are fulfilled in relation to resource utilization. Productivity in general is the total production divided by the total resources used, in this case to produce some specific health care service. The analysis of health system effects can include changes measured economically but the effects can also be analyzed and described qualitatively such as a new process being established or organizational change.

^a^SF-36: Short-Form 36.

^b^PDQ-8: Parkinson's Disease Questionnaire-8.

^c^PSQ-18: Patient Satisfaction Questionnaire Short Form.

^d^GS-PEQ: Generic Short Patient Experiences Questionnaire.

^e^SAPS: Short Assessment of Patient Satisfaction.

^f^WHO: World Health Organization.

^g^ICT: information and communications technology.

#### ICT Interventional Strategies for ICT4PEM

**Figure 2:**
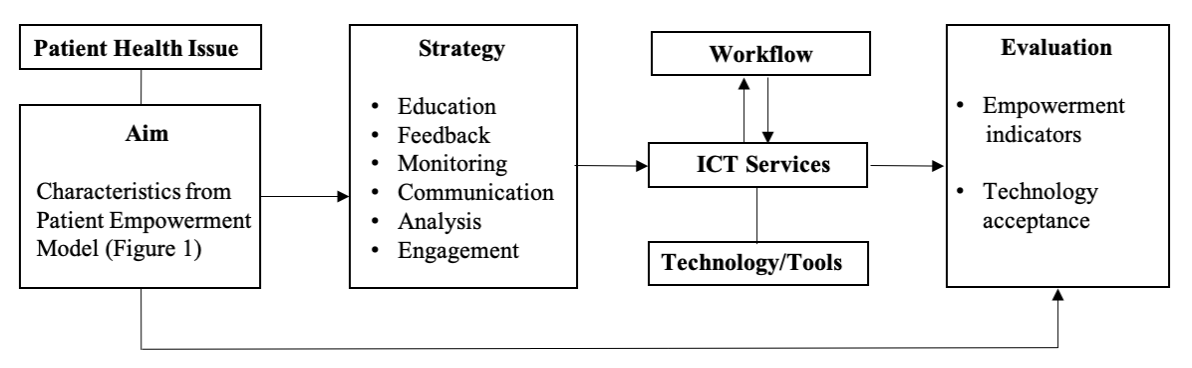
Information and Communications Technology for Patient Empowerment Model (ICT4PEM) framwork.

Using the ICT4PEM framework (Figure 2) requires that an interventional strategy be explicitly selected for influencing the empowerment characteristics that the specific intervention addresses. For the ICT design strategy, our model inherits some elements of the Behavioral Interventional Technology (BIT) model [41]. “Education, Feedback, and Monitoring” are presented as conceptual approaches to achieve behavioral change in the BIT model. The “Engagement” strategy in the ICT4PEM framework corresponds well and combines what is described as “aim setting” and “motivation” in the BIT model to achieve behavioral change. Engagement as an ICT interventional strategy may be coupled to multiple core empowerment characteristics of PEM, such as “Control, Legitimacy, Support, and Coping.” “Communication and Analysis” were added as additional and frequently applied strategies to those already mentioned in the BIT model. “Communication and Analysis” could lead to an improvement in “Knowledge, Legitimacy, and Control” as core PEM characteristics. “Monitoring” disease activities and providing patients with “Feedback” are additional strategies with the potential to improve the PEM characteristics of “Understanding, Control, and Coping.” Taken together, ICT4PEM includes “Education, Feedback, Monitoring, Communication, Analysis, and Engagement” as interventional strategies. The application and proportional contribution of these strategies in the intervention may differ between studies based on the specific context of the patient empowerment intervention. Importantly, the ICT4PEM framework provides precise definitions for each of these strategies in order to enhance conceptual clarity and interoperability. These definitions, along with some important considerations, are summarized in Table 3.

**Table 3:** Definitions of the information and communications technology (ICT) intervention strategy.

Component		Definition
Education		process where an intervention aims to provide the patient with increased knowledge through a defined learning process to empower patients for increasing their understanding of their health situation, characteristics of their disease(s), and participation in shared decision-making concerning the professional health care utilization and for self-care Note 1: Educational strategies may include making knowledge information in text, audio, videos, or interactive learning media available or, alternatively, educational programs where a certain course material is prescribed or required for an individual patient in a certain situation.
Feedback		
	Feedback to the patient	information from the health care system to the patient regarding the present measured health state of the subject that is based on monitoring and presented to the patient at certain intervals or made available for retrieval at the discretion of the patient
	Feedback to the health care system	patient-reported assessment of opinions on the care given at a particular health care organization and possibly an identified health professional
Monitoring		planned and systematic process of observation of a patient’s health status or health care activities, which closely follows a planned course of activities that may include medical technologies and reports to perform the observations Note 1: Health care professionals monitor patients from health care perspectives for optimizing organizational benefits subject to patients’ health status and compares between what is “happening,” what is “expected to happen” and what is intended to happen. Note 2: Monitoring in this context can often serve to allow giving feedback to the patient.
Communication		process where information is flowing between a patient and a health care professional in a bidirectional manner
Analysis		process where collected data are analyzed with a specific health issue in mind and providing the resulting information to a health professional or the patient Note 1: The techniques used for the analyses may depend on evidence-based clinical guidelines where patient specific data is analyzed algorithmically to provide diagnosis or recommendations for treatment, including behavioral changes to be undertaken by the patient. Note 2: Analyses may also be based on machine learning.
Engagement		technique for stimulating the patient to be an active agent for managing their own health

#### Structural Components and Operational Characteristics of the ICT4PEM Framework

The individual components and structure of the ICT4PEM framework are shown in Figure 2. Most of the conceptual elements of the framework are described in detail including operational definitions in the prior two subchapters. The “Workflow, ICT Services, Technologies/Tools, and Evaluation” components of the framework emerged via the expert discussions and later the FGDs. This structure of ICT4PEM represents the integration of the PEM empowerment conceptualization and empowerment characteristics (Figure 1) into the interventional framework and was developed based on the identified specific requirements for an ICT intervention and evaluation specific to patient empowerment targeting. The core patient empowerment characteristics described by PEM correspond to the conceptual aims for the intervention regarding both its targeted design and evaluation. At this stage, at least one or possibly several internal empowerment characteristics should be selected as the primary aims of an intervention whenever an explicit goal is the improvement of empowerment. ICT4PEM also ensures the context specificity of the interventional design by incorporating specific health issues for the patient population that will be the target of the intervention. Health issues are not predefined in the framework. Examples of health issues include, but are not limited to, obesity, diabetes, or Parkinson’s disease.

In the next step, the framework requires a chain-like coupling of each targeted internal PEM empowerment characteristic with one or more “Strategies.” These “Strategies” are described in detail with operational definitions in the previous section. Each selected “Strategy” element should be coupled to specific “ICT Services” with some functionality to support these. Obviously, a given “ICT Service” may be coupled to several “Strategies,” and one “Strategy” may be addressed by several independent “ICT Services”. These services will need to be selected for each specific case and may include a reminder function, set of educational videos, comprehensive display of symptom development to current date, and a messaging service. These services should be delivered by specific “Technologies/Tools” (eg, internet, apps, sensors). ICT4PEM purposely refrains from providing any list and definitions for elements regarding the “ICT Services” and “Technologies/Tools” based on consideration of the rapid technological development that would deem any list soon outdated and the fact that it lies outside the primary goals of ICT4PEM. The framework emphasizes the importance of understanding how the “Workflow” functions in a specific field of application in health care. It includes a consideration of the care plan or how the services will be used in a temporal relation to a symptom or other developments in the course of the patient’s life with the health issue (eg, a service may be designed to mitigate a rare event that may occur for some patients and will only be relevant in such a workflow).

This indicates that an ICT intervention design using ICT4PEM must be considered as a composite of elementary interventions that can be grouped based on the applied ICT tools at the basic level, ICT services at the intermediate level, and ICT strategies at the highest level. Importantly, the framework provides correspondence between intervention and patient empowerment at the basic level, which means that each elementary component of the intervention should target one or several components of patient empowerment with an associated strategy for evaluation. This approach ensures precise, detailed, and conceptually rooted planning and evaluation, along with the possibility for direct comparison between interventional trials.

The framework emphasizes that the intervention project should develop a strategy for “Evaluation” that considers the defined “Conceptual Aims” as primary targets for evaluation. This ensures that the selected PEM empowerment characteristics are in the epicenter of both the interventional and evaluation design. The level of evaluation should reflect and correspond to the level of the interventions. The main rule is that any conceptual component of PEM that is specifically targeted by an intervention should be evaluated. If it is not possible, attempts must be made to explain this deviation and set up the possible link between the intervention and observed changes in the indirect parameters, such as “Empowerment Consequences.” Finally, we included “Technology Acceptance” as an additional indicator for the “Evaluation” domain based on the considerations that no ICT intervention can achieve the desired empowerment unless the technology is used and accepted.

In many cases, an ICT intervention after completion of the first design, implementation, and evaluation would benefit from iterated design to address the possible weaknesses discovered during evaluation. The new round may include changes to the “Strategies,” ICT Services, or various technologies used by these followed by a new evaluation study. ICT4PEM is an overall conceptual framework for the core design process rather than an iterative process.

As described previously, the PEM model features a narrow empowerment conceptualization and, correspondingly, primary targets for the intervention. On the other hand, ICT4PEM does not exclude secondary targets for intervention and evaluation, mostly corresponding to the consequential domain of PEM. As previously explained (in the section ICT Intervention-Specific Requirements for Conceptualization of Patient Empowerment), this distinction between core (perceived) empowerment and empowerment consequences within PEM and its correspondence to the ICT targets are those key components of the framework that help to avoid otherwise inevitable misinterpretation and confusion regarding the interpretation of the results of interventions.

ICT4PEM sets stricter requirements regarding the target of an intervention than the evaluation. For instance, the application of the framework can be applied to the intervention design even if that exclusively evaluates the consequences of empowerment (ie, patient engagement, QoL) with the condition that the primary intervention occurs on the core empowerment as defined by PEM. The theoretical foundation underlying the framework excludes studies from its scope where the sole target of interventions falls within the consequential domain of PEM, even if the evaluation would include the perceptive empowerment in agreement with its definition of PEM. This remains true, even though it is theoretically possible that the intervention on the consequential domain of PEM (ie, messaging patients with encouragement for engagement in the curing process) may indirectly enhance the core (perceptional) patient empowerment as well. However, the framework does not consider such interventions as “empowerment targeting,” although it acknowledges that the wide variety of “side interventions” may affect patient empowerment.

### Case Study Examples

#### Case Study C3Cloud

The C3-Cloud project focuses on elderly patients with diabetes, heart failure, renal failure, and depression in different comorbidity combinations [42]. Three European pilot sites are involved in the study: Osakidetza (Basque Country, Spain), Region Jämtland Härjedalen (Sweden), and South Warwickshire NHS Foundation Trust (United Kingdom). The C3-Cloud system consists of a variety of components: the Coordinated Care and Cure Delivery Platform; Patient Empowerment Platform (PEP); and Clinical Decision Support Module.

The C3-Cloud services include services for both health professionals and patients with multiple conditions. Empowerment was an important explicit goal of the application and the project planning. The PEP provides an internet-based interface for the patients. They can study various educational materials in tailored homework or a possible task program that can entail going through the educational resources selected by the professional for the coming period. The patients are also able and encouraged to review the documented care plan from home, not only once after a scheduled visit but repeatedly as needed. Finally, in the care plan, patients have various tasks to perform such as lifestyle changes regarding exercise, diet, or smoking cessation. These activities should be performed in addition to taking the medication as prescribed during the interaction with the physician and available through the PEP. As stated previously, some patients also have scheduled monitoring tasks that may include daily measurements to be registered into the system and reviewed periodically in another workflow in the life of the patients during the C3-Cloud project.

Here, we illustrate how ICT4PEM could be used to make explicit the various aspects of empowerment in relation to this project.

First, we detail the goals as related to the “Empowerment Characteristics,” noting that the project also has other goals that can be described as empowerment consequences.

Improving patients’ and, when relevant, their informal caregivers’ “Understanding” is a key important objective. The ICT strategy that was selected to improve understanding was “Education” through a set of resources in the form of informative documents and short videos provided through the “ICT Service” PEP, to which the patients had access from home through the internet and a tablet PC as a “Technology.” These educational resources were individually selected by a professional to meet the current stage of disease and the patient’s presumed ability. The “ICT Service” had a large set of educational resources for each of the four disorders we targeted, and patients had two or more of these disorders.

Another strategy selected to improve understanding was “Feedback.” In this case, this means that the professional care plan with results such as lab tests and planned medication and other actions is available to the patients through the “ICT Service” PEP. For some patients, we also selected “Monitoring” as a strategy, where the patients could automatically register certain measurements such as weight or blood pressure using special devices as “Technology” at home, into the system through the PEP interface to the system. The results then became available to the professionals as well to the patients.

Finally, “Communication” was a strategy to increase feelings of “Support” as another major characteristic and to some extent to exert “Control” as another important empowerment characteristic. This was implemented through a “Messaging” ICT service, being part of the PEP.

The different ICT services for the patients to improve empowerment has a special relationship to the “Workflow” of the patient care processes. The start of the use of the system comes after a scheduled visit to the primary care physician where the current state of the chronic diseases was evaluated and a care plan was drawn up using automatic decision support for the health professionals based on the patient data and available clinical guidelines. This aspect targets the professional user, but the patient is of course also benefitting from hopefully improved quality of care through this support service.

There are a number of “Empowerment Consequences” that are possible results of the C3-Cloud project and that will be evaluated during the project: HRQoL, “Patient satisfaction,” “Clinical Outcomes,” and “Health Care System Effects.”

HRQoL, as measured with the EQ-5D, could potentially be affected, although the chronic nature of the selected diseases and the relatively small number of patients in our study means we may not detect it in this study.

“Patient Satisfaction,” defined as “the degree of fulfilment of the patient’s expectation of the health care services received” will be assessed using our own instrument rather than using tools such as the Patient Satisfaction Questionnaire Short Form (PSQ-18), Short Assessment of Patient Satisfaction (SAPS), or Generic Short Patient Experiences Questionnaire (GS-PEQ).

“Clinical Outcomes” will be measured and compared to a control group from the same regions receiving the same care in principle but without the C3-Cloud intervention.

“Health Care System Effects,” defined as “the health care system resource utilization before, during, and after a specific new procedure such as an ICT-based patient empowerment intervention” will be partly measured through interviews with professionals and decision makers and also by a calculation of potential economic consequences if the project should be launched on a large scale either throughout the regions or nationally.

An assessment program has been planned where patients’ use and views on the various components are evaluated using questionnaires. These are both related to “Technology Acceptance” but can also be seen as addressing empowerment even if the explicit goals as stated here are not mentioned in the design of these questionnaires.

#### Case Study EMPARK

To explain the ICT4PEM framework, we provide another example of a system (EMPARK) aimed at improving the treatment of Parkinson’s disease by empowering patients with data from their own measurements [43]. EMPARK is an Internet of Things–based system designed to help patients improve their self-management and increase their self-awareness. The system consists of sensors for logging motor function and sleep information, an electronic dosing device for logging and delivering dose intakes, and a tablet-based app for logging daily activities, meal timing, and self-assessments from the patients’ home environments. A separate tablet-based app feeds the gathered information back to the patients, providing them access to their individual symptoms and activity records. Additionally, treating clinicians gain access to the detailed data of their own patients through a web application. The overall objective of EMPARK is to promote patient empowerment and thus improve patients’ HRQoL.

Using the ICT4PEM framework, the primary “Aims” are first defined from the “Empowerment characteristics,” which in this case will be to provide “Understanding” and “Control” to the patients. The selected “Strategies” consist of “Monitoring” and “Feedback” for collecting and visualizing the data, respectively. The “ICT Services” include the collection of sensor-based and patient-reported data as well as the patient interface where the data is presented in an easy-to-understand and comprehensive manner. These services are intended to be used in routine clinical practice to allow patients to take an active role in the decision-making process and improve the patient-clinician interaction (“Workflow”). The services are supported by “Technologies/Tools” such as the sensors, custom applications, and communication architecture. The “Evaluation” of the intervention using the EMPARK system focuses on the extent of achieving the empowerment characteristics, which are “Understanding” and “Control” and “Technology Acceptance,” which has been studied separately [44]. Possible “Empowerment” consequences of the EMPARK system include: “HRQoL Outcomes,” as measured by EQ-5D and Parkinson’s disease-specific instruments (eg, the 39-item Parkinson’s Disease Questionnaire); “Patient Satisfaction,” as measured through customized interviews; “Patient Engagement,” as measured by analyzing systems data (eg, compliance with measurements using the sensors); “Patient Adherence,” as measured by analyzing systems data (eg, adherence to the treatment plan); and “Clinical Outcomes,” as measured by Parkinson’s disease–specific clinical rating scales (eg, Unified Parkinson’s Disease Rating Scale).

## Discussion

The objective of our study was to develop a framework for ICT interventions that aim to empower patients. To the best of our knowledge, the ICT4PEM is the first framework model that pursues this goal and was developed to facilitate and provide guidance for designing, implementing, and evaluating ICT interventions on patient empowerment. The ICT4PEM systematically addresses an array of challenges that investigators commonly face during the design of the ICT interventions.

Improving patient empowerment via ICT-based interventions is a commonly accepted strategy, but the evaluability and real scientific value of these efforts have remained elusive largely due to conceptual and methodological limitations. Consequently, it frequently remains unclear what exactly was targeted and how the effect was evaluated in case of specific ICT interventions on patient empowerment. Previous conceptual models of patient empowerment have limited value to be utilized as a framework or patient empowerment conceptualization model for ICT interventions. Early patient empowerment models were characterized by narrow contextuality that limited their applicability outside a specific clinical scenario or disease setting [12]. Comprehensive and synthesizing conceptual models of patient empowerment were recently published [12,45,46]. The model by Bravo et al [12] is the latest and most comprehensive that positions patient empowerment in a wider contextual perspective by elaborating psychological, medical, or social constructs that conceptually surround patient empowerment [12]. In our view, the possible contribution of this model to ICT intervention design targeting patient empowerment is in its ability to reveal potential associations and secondary targets for the intervention. Although the model by Bravo et al [12] may help to identify potential conceptual elements of patient empowerment for an ICT intervention design, it lacks definitions for the included conceptual elements of empowerment and allows speculations regarding the described parameters being part or falling outside the scope of the patient empowerment conceptualization. Although ICT4PEM utilizes some elements of the patient empowerment model of Bravo et al [12] in the identifications of potential interventional targets, ICT4PEM contrasts that model by several means. Our framework, and specifically its empowerment conceptualization model (PEM), provides clarity with ISO-style definitions and clear separation of core characteristics of patient empowerment and consequential domains. In contrast to prior patient empowerment models, which largely inflated or contextually limited the conceptual boundaries and components of patient empowerment, the ICT4PEM framework purposely narrows the conceptual boundaries of patient empowerment to the core, perceived characteristics from the patient-centric approach. This is based on both theoretical and practical considerations. The theoretical considerations include the deep patient centeredness of empowerment and the inherited difficulties to establishing a commonly accepted definition and conceptual boundaries. In contrast to prior synthesizing approaches, we turned our attention to studies that provided pure patient-derived conceptualization by conducting patient interviews. It helped us to elaborate PEM, a patient-derived empowerment self-conceptualization with minimal modifications. Consequently, PEM exclusively includes patient perceptions as core empowerment, and behavioral domains of prior empowerment models are instead grouped together with other parameters as patient empowerment consequences. Providing definitions in the ISO style for each conceptual element both within the core empowerment and consequential domains of the PEM further facilitates the precise targeting and evaluation of the intervention. We argue that these features of the ICT4PEM are key to promoting future interoperability and comparison of the results between ICT interventions on patient empowerment.

It is generally assumed that improving patient empowerment translates into wider benefits for both the patients and health provider, such as better quality of life, disease control, or more effective health care utilization. However, there is subtle evidence to support this notion. ICT interventional trials using the ICT4PEM framework can now provide the possibility to quantitatively analyze the association between core patient empowerment and possible consequences, such as changed patient behavior, disease management and control, quality of life, and health care utilization.

The ICT4PEM framework provides ICT interventions with the methodology to link interventional objectives, strategies, ICT services, and evaluation centered on a clarified conceptualization of patient empowerment by PEM. The ICT4PEM framework explicitly sets patient empowerment and its consequences as the objectives for the elaboration of the selected strategies and ICT services. Technically, it means the requirement for allocating one or several specific ICT interventional steps to one or several PEM core characteristics for their modification. This design also ensures a detailed inventory of ICT interventions, where the elementary ICT components are defined at the level of the technology and ICT tool used and grouped further based on the ICT service type, ICT strategy, and specific intervention, respectively. With respect to the included list of ICT strategies and services, ICT4PEM benefits from prior eHealth-related ICT frameworks. The framework follows the logic of the BIT [41] in two specific ways. First, BIT, similarly to the core concept of ICT4PEM, makes a distinction between conceptual and instantiation domains. Second, it also describes ICT developments as a chain of developmental phases, with each having a well-defined specific target at the conceptual level. However, in contrast to ICT-PEM, BIT defines broadly all ICTs that support users in changing behaviors and cognitions related to health, mental health, and wellness, while not addressing the conceptual controversies of patient empowerment. Another difference between ICT4PEM and BIT is that ICT4PEM does not intend to provide a detailed recipe for the technological instantiation of each ICT intervention step, as it is unnecessary for the overall goal and would pose unnecessary limitations to its applicability.

Although ICT4PEM demands the inclusion of the targeted PEM core characteristics into the evaluation, it refrains from providing methodological recommendations for the evaluation. Instead, it extends the wider applicability of the framework by suggesting that the exact methodology of evaluation should follow the specific interests of the study. However, the framework specifically addresses two important issues. One, as it was previously explained, is the strong recommendation for the inclusion of the targeted PEM core characteristics into the evaluation. The other consideration is technology acceptance. The role of technology acceptance in the efficacy of ICT interventions is well known and described in the literature [47,48]. More specifically, it is one of the major sources of possible bias in the observed results based on the characteristic of the study population. We warrant that ICT4PEM should be applied in conjunction with well-established frameworks, such as the Technology Acceptance Model [49], during the evaluation phase of the ICT4PEM, in order to mitigate such biases.

The application of ICT4PEM was demonstrated by showing how it could be functional in the design and evaluation of two ongoing, complex ICT interventions with different health status backgrounds: C3Cloud and Empark. What is common in these ICT interventions is their incorporation of patient empowerment as a major target, besides other goals. We emphasize the real-life aspect of our examples, as ICT interventions on empowerment are rarely limited to empowerment, but rather are complex regarding their background (ie, health status, workflow) and desired effects. Although our example projects were not designed by ICT4PEM, our analyses clearly identify the applicability of the framework, as it was possible to identify an array of individual ICT interventional steps with clear targets within the PEM core or consequential characteristics. During the utilization of ICT4PEM in their later, evaluation phase, these projects could benefit most from (1) clear definitions for empowerment characteristics by ICT4PEM, (2) establishing the proportional contribution of the single interventions to change in a given parameter, and (3) establishing possible causative relationships between the observed changes of the parameters regardless of their original relative importance as a target. The first point is a straightforward advantage in light of the current confusion with respect to the patient empowerment conceptualization. The latter two points need explanation. ICT4PEM anchors the interventions to the workflow of health provision and consequently enable the adjustment and harmonization between the temporality of the evaluation and interventional steps. For example, investigators may identify the improvement of understanding and control (as core PEM characteristics) to be a cause of observed improvement in patient engagement and HRQoL (as consequential PEM characteristics) and not the other way around, by establishing the temporality of these changes. This could be done by sequential interventional and evaluation steps designed to fit the specific workflows of the health care environment in which the study is implemented.

### Limitations

The ICT4PEM framework has some limitations. Most importantly, it has not yet been used to guide complex ICT interventions starting from the design to the evaluation. This warrants the real-life validation of ICT4PEM. Although we demonstrated that ICT4PEM can be used to guide empowerment-targeted strategies within very complex interventions, the full complexity of ICT intervention projects may reveal some specific design and evaluation issues that ICT4PEM does not cover. There are ample other conditions for the overall success of any ICT intervention in health care that are beyond the focus of our framework. We are aware that successful ICT project implementation in health care depends on several other crucial conceptual elements and practical considerations as well and that all should be taken into account. ICT4PEM provides a vital conceptual background and requirements design for patient empowerment but we suggest our model be used together with broader models of ICT implementation within health care. For instance, the CeHres Roadmap by van Gemert-Pijnen et al [50] is an established and well-cited model with a holistic approach to eHealth project implementation in general.

Applicability of ICT4PEM for interventions that only marginally address patient empowerment and set other parameters as their main target may instead benefit from other ICT models, such as the BIT [41]. Finally, we acknowledge that ICT4PEM does not address the problems of patient empowerment operationalization. Specifically, the framework could not include instruments on how to measure core empowerment as well as consequences for specific situations due to the limitations of the quantity and quality of the published literature on this issue. Further research is needed to validate our framework and to provide examples on its applicability in different diseases, health care and ICT intervention settings.

### Conclusions

The new framework, ICT4PEM, with newly proposed definitions of core characteristics of empowerment and empowerment consequences, can be useful for the design of ICT interventions targeting empowerment and can assist the development of methods to measure the results in this dimension. However, further evaluation in future interventional studies are required to assess the generalizability of the model.

## Abbreviations

BIT: Behavioral Interventional Technology
eHealth: electronic health
FGD: focus group discussion
GS-PEQ: Generic Short Patient Experiences Questionnaire
HR-QoL: health-related quality of life
ICT: information and communications technology
ISO: International Organization for Standardization
ICT4PEM: ICT for Patient Empowerment Model
PDQ-8: Parkinson's Disease Questionnaire-8
PEM: patient empowerment model
PEP: Patient Empowerment Platform
PSQ-SF: Patient Satisfaction Questionnaire Short Form.
QoL: quality of life
SAPS: Short Assessment of Patient Satisfaction
SF-36: Short-Form 36
WHO: World Health Organization
